# A Natural Language Processing Approach to Identify Negative Patient Descriptors in Electronic Health Records for Maternal Care

**DOI:** 10.1055/a-2703-7227

**Published:** 2025-10-28

**Authors:** Azade Tabaie, Angela D. Thomas, Emily K. Mutondo, Allan Fong

**Affiliations:** 1 Center for Biostatistics, Informatics, and Data Science, MedStar Health Research Institute, Columbia, Maryland, United States; 2 Department of Emergency Medicine, Georgetown University Medical Center, Washington, District of Columbia, United States; 3 Healthcare Delivery Research, MedStar Health Research Institute, Washington, District of Columbia, United States; 4 Georgetown University School of Health, Washington, District of Columbia, United States

**Keywords:** maternal health, natural language processing, machine learning

## Abstract

**Background:**

Maternal harm, especially for Black women, is a significant health care issue. Unstructured clinical notes in electronic health records (EHRs) may reveal unsafe maternal care. Prior studies using natural language processing (NLP) have shown that tone and sentiment in notes contribute to preventable safety events.

**Objective:**

This study aimed to examine whether negative patient descriptors in EHR clinical notes are associated with adverse maternal outcomes and how their use varies by patient demographics.

**Methods:**

We conducted a retrospective cohort study of women who delivered at two large birthing hospitals in Washington, DC between January 1, 2016 and March 31, 2020. Using a predefined list of negative keywords (e.g., combative) and NLP, we identified sentences from clinical notes for manual review. Two subject matter experts labeled keywords as “negative descriptors” if they negatively described patients. A logistic regression model with elastic net regularization was trained on the labeled sentences to classify the remaining corpus. We evaluated the prevalence of negative descriptors by race, age, insurance type, and pregnancy outcomes, and calculated adjusted odds ratios.

**Results:**

Among 190,026 clinical notes from 9,302 patients, 719 notes associated with 444 patients contained at least one negative descriptor. Of these, 313 (70.5%) were Black, 45 (10.1%) were White, and 86 (19.4%) were from Other racial groups ( *p* < 0.001). Negative descriptors were more common among younger patients (18–29 years: 49.3%) and those with Medicare/Medicaid insurance (65.3%). Although case patients—defined as those with postpartum readmission or severe maternal morbidity—had slightly fewer descriptors overall, they had higher adjusted odds of having them. Black patients were associated with higher odds, and commercial insurance with lower odds, of having negative descriptors.

**Conclusion:**

Negative descriptors appear disproportionately in the notes of Black patients and those with public insurance, suggesting implicit bias in documentation. Addressing biased language is essential for improving equity in maternal care.

## Background and Significance

Racial disparities in maternal outcomes represent a pressing patient safety crisis in the United States. Maternal harm is a major crisis that disproportionately affects Black women. 1 In a Centers for Disease Control and Prevention (CDC) study, based on analysis of national data on pregnancy-related mortality, Black women were three times more likely to experience maternal harm than White women. 2 Studies using in-depth severe maternal morbidity reviews found that the most frequent preventable factors were provider-related and/or system-related, including substandard provider practices, delay, or failure in diagnosis or recognition of high-risk status, and delay or inappropriate treatment. 3 These provider-related and system-related contributing factors disproportionately affected historically minoritized groups. 4 With these facts: (1) high rates of maternal harm, (2) Black women at significantly higher risk of maternal harm, (3) most maternal harm being preventable, and (4) providers and systems often contributing to that harm—this preventable maternal harm crisis is better defined as a patient safety crisis and more specifically, a maternal safety crisis that disproportionately affects Black women.

In this study, we define maternal harm as preventable adverse health outcomes that occur during pregnancy, childbirth, or the postpartum period—particularly those resulting from provider- or system-level failures such as missed diagnoses, inappropriate clinical decisions, or failures in communication. This framing aligns with national patient safety efforts, which recognize that a large proportion of maternal deaths and severe maternal morbidity events are avoidable through timely and equitable care.

### Racial Biases and Patient Safety

A successful patient safety science approach relies on the rigorous analyses of several clinical and administrative data sources to uncover patient harm events, such as administrative claims data, patient safety reporting systems, risk management data, and medical record abstraction. 5 Patient safety reporting system is a process where health care professionals report incidents, near misses, and other safety concerns to improve patient care and prevent future harm. The safety incidents can range from medication error to communication failure and patient fall. These traditional patient safety reporting systems each have value, yet are not without challenges. For example, previous research has found that in patient safety reporting systems, health care staff are significantly more likely to report harm and near-miss events for White Patients as compared with Black patients. 6 7 This differential reporting by patients' race leads to disparities in learning from patient safety events and potentially contributes to disparities in harm. 6 7 Consequently, relying on traditional patient safety reporting surveillance alone will not address the disproportionate harm that Black women face.

Racism, discrimination, and implicit bias contribute to adverse health outcomes, including adverse maternal outcomes. 8 9 10 11 12 13 14 15 16 Biased documentation in electronic health records (EHR) may serve as both a marker and a mechanism of unsafe care. The Giving Voice to Mothers Study found that women of color are more likely to experience mistreatment during the perinatal period. 15 These mistreatment experiences most often included being shouted at, scolded, threatened, ignored, or receiving no response to requests for help. 15 Black mothers have also reported experiencing stereotypes stigmatizing Black motherhood, including assumptions about being single, low income, poorly educated, and having multiple children, which can influence the quality of care delivered. 17 Research has shown that Black women are less likely to have their symptoms believed, more likely to be described using stigmatizing or behavioral language in medical notes, and less likely to receive adequate pain management or respectful communication. These disparities are not due to patient behavior or clinical need, but rather reflect the effects of institutionalized racism, interpersonal discrimination, and provider implicit bias across the care continuum. 18 19 20 21

In this way, biased language is not merely a reflection of provider bias—it is also a contributor to downstream patient safety risks, particularly for historically marginalized populations. These types of experiences are often missed in traditional patient safety reporting systems that rely on administrative coding and reports by health care staff. Our study builds on this patient safety framework by using natural language processing (NLP) to identify biased documentation that could contribute to inequitable and potentially unsafe care trajectories.

### Negative Language in the Electronic Health Record Documentation

There has been a growing focus on how physicians document clinical notes in EHR. A qualitative study found that physicians express negative and positive attitudes toward patients when documenting EHR clinical notes, which potentially reflect bias and affect the quality of care provided to patients. 22 Therefore, EHR can be useful to identify opportunities for improving patient safety. EHR data may contain signals for unsafe maternal care delivery. Prior studies have analyzed unstructured clinical notes in EHRs using NLP to detect tone and sentiment. For example, Cohan et al. demonstrated that negative tone in physician documentation was associated with increased risk of preventable adverse events and diminished quality of care by analyzing sentiment-laden phrases across thousands of clinical notes. 23 24 Another study further validated that NLP methods can be used to identify linguistic cues associated with clinician frustration, bias, or lack of empathy. 25 These studies highlight that EHR documentation can serve as a signal for patient safety and quality issues and informed our hypothesis that negative descriptors in maternal health notes may reflect underlying provider bias. Building on this prior work, our study focused specifically on maternal patients and sought to identify racially patterned use of stigmatizing language in delivery-related notes.

It is important to note that the language we refer to as “negative descriptors” is not inherently inappropriate or stigmatizing. In some cases, these terms may be clinically justified or necessary to document observable behaviors, symptoms, or care challenges that are relevant for diagnosis or treatment. For example, describing a patient as “agitated” may alert clinicians to assess for underlying conditions such as hypoglycemia, hypoxia, or adverse drug effects. Our aim is not to suggest that these words should never be used, but rather to examine whether their use disproportionately affects certain patient groups, particularly along racial lines, in ways that could reflect or reinforce inequities.

### Objectives

In this study, we analyzed potentially negative descriptors in the unstructured clinical notes in EHR data recorded for women who had a delivery encounter at one of two large, birthing hospitals in Washington, DC.

## Methods

### Data

#### Inclusion Criteria

The cohort was defined as female patients who had delivery encounter at one of two large, birthing hospitals in Washington, DC, from January 1, 2016, to March 31, 2020. All patients included in the cohort had documented delivery encounters, thereby ensuring they were within childbearing age. Patients with Medicare insurance were presumed to be enrolled due to disability or special qualifying conditions rather than advanced maternal age. The features from EHR data included person and visit encounter identifiers, age at the time of delivery, race, insurance type, unstructured free-text clinical notes, which were recorded during the delivery or postpartum encounters. For this study, we only included clinical notes that were written during the delivery hospitalization or during the initial postpartum hospital stay. Notes recorded after a postpartum readmission were excluded from the analysis to ensure that all extracted language preceded or coincided with the index adverse event. Five clinical note types were selected: history and physical note, review of systems documentation, history of present illness documentation, physical examination documentation, triage note. Patients were included in the analysis if the demographic information and at least one of the five types of history-related clinical notes were recorded for them. Patient delivery encounters were further categorized into two groups for this study: (1) cases and (2) controls. Case group included patients who experienced severe maternal morbidity and/or had postpartum readmission within 42 days of delivery. Case group included patients who experienced severe maternal morbidity and/or had postpartum readmission within 42 days of delivery. Maternal morbidity was determined through CDC's 21 severe maternal morbidity indicators, which include a list of diagnosis and procedure codes based on ICD-10-CM and ICD-10-PCS coding systems. 26 Control group included patients who did not experience a severe maternal morbidity event or postpartum readmission. To further ensure that the control group reflected low-risk deliveries, we also excluded patients with outcomes indicative of clinical complications, including low birthweight (<2,500 g), preterm delivery (<37 weeks' gestation), instrument-assisted delivery (vacuum or forceps), and neonatal intensive care unit admission. These additional criteria were intended to reduce potential confounding and provide a cleaner contrast to the case group, which included patients with adverse maternal outcomes.

#### History and Physical Electronic Health Record Clinical Notes

**Fig. 1:**
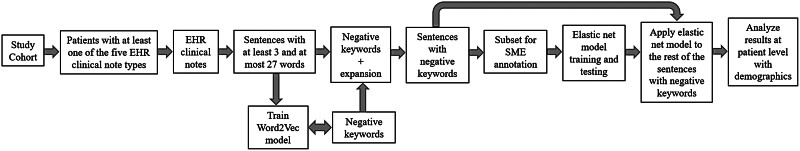
Summary of the methods. The diagram on the left presents the inclusion criteria based on five her clinical note types. Word2Vec models were trained to expand the list of negative keywords. The diagram on the right demonstrates validating the sentences with negative keywords by our subject matter experts (SMEs). A machine learning classifier was trained on the reviewed sentences, then tested on the rest of the sentences to detect all the sentences with negative keywords. Finally, patient-level data were analyzed to compare the demographics of the patients who had a recorded negative keyword with the rest of the cohort.

We utilized clinical notes that contained information regarding history and physical condition of a patient (e.g., history of present illness documentation, review of systems documentation, physical examination documentation, history and physical, and triage note). Such clinical notes contain a comprehensive narrative about a patient captured in a provider's language. Moreover, other providers extract relevant information from history and physical-related notes to include in their own chart notes, such as progress notes or discharge summaries. General free-text data preprocessing was applied on the clinical notes. Fig. 1 presents an overview of the methods used in this study.

#### Ethical Considerations

The original data collection and study protocol were approved by the Institutional Review Board (IRB number 00005269). A waiver of informed consent was granted by the IRB based on the study's minimal-risk classification. All identifiable fields of the structured EHR data were removed after data pull, which was in accordance with the HIPAA Safe Harbor method. Data was analyzed within a HIPAA-compliant secure enclave environment to ensure privacy and security throughout the research process.

### Negative Descriptors

#### Negative Keywords from a Prior Study

In a prior study, Sun et al. incorporated clinical notes to investigate how racism and bias may be communicated in the medical records. 27 The authors selected 15 negative keywords to detect potentially negative descriptors in recording EHR clinical notes: nonadherent, aggressive, agitated, angry, challenging, combative, noncompliant, confront, uncooperative, defensive, exaggerate, hysterical, unpleasant, refuse, and resist. 27

#### Expanding Search

We use two NLP techniques to expand the negative keyword searches. First, we adjusted the negative keywords to permit detection of alternative grammatical forms. To that end, we utilized the root of a negative keyword instead of the keyword itself (e.g., “aggress” instead of “aggressive”). Supplementary Appendix A (available in the online version only) includes the complete list of 15 negative keywords' roots.

In addition, words that are semantically relevant to the negative keywords can be employed to identify negative descriptors in clinical notes. To find semantically relevant words to the negative keywords, we looked at other words presented in the clinical notes, which shared the same root as the negative keywords. To that end, the clinical notes were scanned to find other negative keywords with the keywords' roots. If the most frequent identified words for a negative keyword's root was not the keyword itself, the more frequent identified word was replaced by the negative keyword in the list. We used the final list of negative keywords in the next step.

**Fig. 2:**
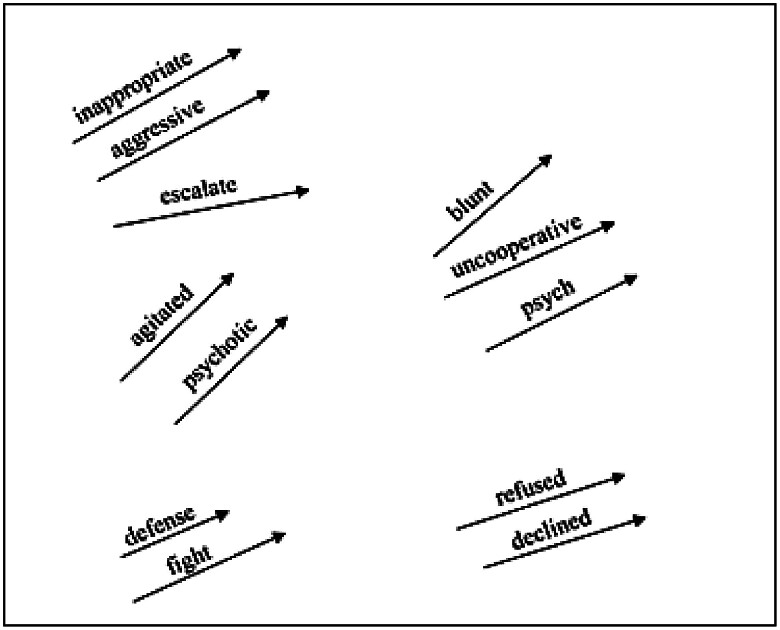
Word2Vec. The figure presents how Word2Vec model find semantically relevant words in free-text data.

We utilized Word2Vec model on sentences from clinical notes to find semantically relevant words to the negative keywords. 28 Using Word2Vec model, each word can be represented as unique numerical vector. Numerical vectors representing semantically similar words have small distances to each other. For instance, there is a small distance between the vectors representing *agitated* and *psychotic* in Fig. 2 . To expand negative keywords in this study, we trained Word2Vec models on our EHR clinical notes data and employed the trained models to find words similar to the identified list of keywords. We trained two Word2Vec models with both skip gram and continuous bag of words training algorithms. In both models, the window length (i.e., the distance between a target word and words around the target word) was set as 5. The minimum occurrence was one; therefore, all words were included in training the Word2Vec models regardless of their frequency in the clinical notes.

The words identified by the Word2Vec models were presented to two subject matter experts (SMEs) with expertise in social determinants of health, implicit bias, and clinical language. These experts served as the reference standard for evaluating whether the identified terms were semantically and contextually aligned with the concept of a negative patient descriptor. Each candidate word was manually reviewed, and only those validated by both reviewers as plausible negative descriptors were retained in the final list. This expert validation process served as the ground truth for refining and expanding our keyword set. Using Word2Vec, we identified 225 additional expansion terms. In total, 97 (43.1%) of these words were identified as semantically relevant negative keywords by the SMEs. Table SB-1 in Supplementary Appendix B (available in the online version only) includes all the words similar to the negative keywords identified by Word2Vec models. Table SB-1 was presented to our SMEs to be validated. Supplementary Appendix B (available in the online version only) also presents the validated similar terms that were added to the list of negative keywords. Negative keywords such as agitated, aggressive, angry, and noncompliant had the greatest number of validated similar words.

The approved semantically relevant words from Word2Vec model along with the list of negative keywords' roots were employed to find the sentences containing the negative keywords in clinical notes.

### Identifying Negative Keywords in Clinical Notes

The dataset included 190,026 clinical notes, with one of the five selected note types: history and physical note, review of systems documentation, history of present illness documentation, physical examination documentation, triage note from delivery and postpartum encounters. These data represented 87,613 visit encounters associated with 9,302 unique patients.

Sentences with fewer than three words and more than 27 words (the 95 ^th^ percentile for sentence length in our corpus) were excluded from analysis. Although keywords were the primary unit of interest, preliminary manual review revealed that very short sentences often lacked sufficient context to determine whether the keyword described the patient, while very long sentences often contained multiple clauses, clinical impressions, or templated text that made semantic interpretation and annotation ambiguous. Filtering these edge cases helped improve the clarity of expert annotation and the precision of the downstream classification model. This decision also aligns with common preprocessing practices in real-world NLP pipelines. 29 Setting the threshold for the number of words in a sentence led to a final number of 3,916,248 sentences from the 190,026 notes. In total, 469,486 sentences (out of 3,916,248, 12.0%) had at least one negative keyword. These sentences were associated with 1,384 clinical notes representing 851 unique patients. To lower the burden of manual review of the sentences, the initial scan of the sentences with negative keywords was conducted. Among the 469,486 sentences with negative keywords, there were common sentences, which repeated multiple times (e.g., patient was alert and cooperative) or clearly did not convey negative patient description (e.g., a 35-year-old pleasant patient). Such sentences were removed from the list of sentences for further review.

In total, 1,874 sentences (0.40%, out of 469,486) were selected in a stepwise approach and annotated by two reviewers with expertise in social determinants of health, health disparities, and clinical workflows and operations to utilize in training machine learning classifiers. Supplementary Appendix C (available in the online version only) demonstrates examples of sentences in clinical notes, which were validated to have a negative descriptor.

Further, we assessed whether the annotated subset of 1,874 sentences was demographically representative of the broader corpus of 469,486 sentences containing negative keywords. Specifically, we compared the patient-level distributions of age, race, and insurance type between the two sets. The distributions were similar across groups, although the annotated sample showed a slightly higher proportion of patients with commercial insurance. These comparisons support that the annotated sample, while smaller, reflects the broader data patterns and demographic context of the full corpus.

### Models

#### Sentence Annotation

The identified sentences containing negative keywords were presented to the two reviewers. Reviewers annotated each sentence as either “negative descriptor” or “not negative descriptor.” Negative descriptor is defined as using a negative keyword to explicating describing the patient by negative adjectives or behavior (e.g., aggressive patient). The clinical experts' review lead to one of the following three labels: (1) negative descriptor, (2) not negative descriptor, or (3) out-of-context (negative keyword was not used to describe a patient). This manual review also accounted for negated or contextually neutral uses of keywords. For example, sentences such as “patient is not combative” were labeled as “not negative descriptor” or “out-of-context.” In this way, negation was handled implicitly through expert annotation. There were 11 (out of 1,874) sentences for which the two reviewers disagreed on the presence of a negative descriptor. The disagreements were around the patient's compliance to the treatment and behavior. To maintain labeling precision and avoid introducing ambiguous or subjective examples into the training data, these sentences were conservatively labeled as “out-of-context” and excluded from modeling. While we did not conduct a formal consensus adjudication process for these specific cases, our conservative labeling approach prioritized data quality for later classifier development. Cohen's Kappa score was 0.7, which indicated the level of agreement between the two reviewers.

The manually reviewed sentences were split to training and validation set (80%) and testing set (20%) for the modeling process. The number of annotated sentences was constrained by the time-intensive nature of expert manual review, particularly given the required expertise in social determinants of health and clinical documentation. To maximize representativeness, we used a stepwise selection strategy that prioritized diversity in sentence structure and content and excluded commonly repeating phrases unlikely to reflect bias. Nonetheless, we recognize the potential for misclassification bias due to unreviewed edge cases, which we further acknowledge as a limitation in the discussion.

#### Machine Learning Classifiers

Binary logistic regression model with elastic net regularization (elastic net model) and 5-fold cross-validation was trained to classify sentences with none versus at least one negative descriptors. All the sentences utilized in training the elastic net model had a negative keyword. Elastic net or L1L2 regularization is a weighted combination of Least Absolute Shrinkage and Selection Operator (LASSO or L1) and Ridge (L2), which is a model well-suited for classification tasks with high-dimensional feature space, such as text classification. 30 31 For performance comparison, other machine learning classifiers were also trained: simple logistic regression model, random forests, and support vector machines. We used Bayesian optimization and grid search during training to mitigate the overfitting and improve generalizability for all trained classifiers. The performance of the classification models was calculated in terms of area under receiver operating characteristics curve, sensitivity, specificity, positive predictive value, negative predictive value, F-1 score, accuracy, and area under precision–recall curve. The best performing model was the elastic net model, which was then applied to the rest of the unlabeled sentences with negative keywords to classify the sentences into the ones with none or at least one negative descriptors.

### Study Variables and Statistical Analysis

After classifying the rest of the sentences with negative keywords, logistic regression models were trained to find the odds of having a negative descriptor recorded in a patient's clinical notes as a function of the demographic features (i.e., age, race, and insurance type) and the pregnancy type (i.e., case or control group). To preserve temporal validity, predictors (i.e., presence of negative descriptors) were drawn exclusively from the index delivery encounter, while outcome status (case versus control) was based on whether patients subsequently experienced a severe maternal morbidity event or were readmitted within 42 days. Notes from any readmission encounters were excluded from text analysis. Adjusted and unadjusted odds ratios were calculated.

## Results

### Study Population

There were 9,776 unique patients who had delivery encounters during the study period. Out of which, 9,302 patients had at least one of the selected five clinical notes. A total of 851 (9.15%, out of 9,302) patients had at least one negative descriptor recorded in clinical notes. Among these 851 patients, 315 patients (37%) were represented in the subset of 1,874 annotated sentences. Table 1 presents the demographics for the subset of the 315 patients. Demographic characteristics in this subset remained similar to the broader population with negative keywords, with slightly higher proportions of patients with commercial insurance, consistent with the overall distribution of negative keyword usage in the full corpus.

**Table 1:** Patients' characteristics

	All patients with negative keywords		With negative descriptor		Without negative descriptor	
	%	Number	%	Number	%	Number
Number of patients	100	315	16.8	53	83.2	262
Number of clinical notes	100	1686	22.8	385	77.2	1301
Age (y)						
18–29	36.5	115	43.4	23	36.2	95
30–44	61.3	193	62.3	33	61.1	160
45+	2.22	7	0	0	2.7	7
Race						
Black	61.9	195	77.4	41	58.78	154
White	21.9	69	13.2	7	23.66	62
Other	16.19	51	9.43	5	17.56	46
Insurance type						
Medicare/Medicaid	45.7	144	62.3	33	42.4	111
Commercial	53.0	167	35.8	19	56.5	148
Self-pay	1.27	4	1.89	1	1.15	3

Notes: This table outlines the characteristics of patients who had annotated clinical notes with keywords. The cohort is divided based on the decision of the two subject matter expert reviewers as the ones with negative patient descriptor or not.

### Performance of Machine Learning Models

Supplementary Appendix D (available in the online version only) includes the list of selected features from the sentences of the clinical notes to train the classifiers, along with their associated estimated coefficient. The performance of the classification model is presented in Table 2 . The classification model achieved a high F-1 score of 0.74 on the testing data, which indicates the strong performance of the classification model in classifying negative descriptors among the rest of the sentences with negative keywords from the clinical notes in our study.

**Table 2:** Performance metrics of the negative keywords classifiers

	Elastic Net		Logistic Regression		SVM		Random Forests	
Metrics	Train data	Test data	Train data	Test data	Train data	Test data	Train data	Test data
AUROC	0.99 [0.98, 0.99]	0.89 [0.86, 0.94]	0.88 [0.87, 0.9]	0.79 [0.79, 0.8]	0.91 [0.89, 0.94]	0.80 [0.79, 0.82]	0.93 [0.90, 0.94]	0.79 [0.79, 0.82]
Sensitivity	0.81 [0.81, 0.83]	0.61 [0.58, 0.61]	1.00 [0.81, 1.00]	1.00 [0.61, 1.00]	0.82 [0.81, 1.00]	0.61 [0.58, 1.00]	0.87 [0.81, 0.90]	0.58 [0.58, 0.65]
Specificity	0.99 [0.99, 1.00]	0.99 [0.99, 1.00]	0 [0, 0.99]	0 [0, 1.00]	0.99 [0, 1.00]	0.99 [0, 1.00]	0.99 [0.99, 1.00]	0.99 [0.99, 1.00]
PPV	0.91 [0.88, 0.98]	0.95 [0.90, 1.00]	0.08 [0.08, 0.91]	0.08 [0.08, 0.95]	0.98 [0.08, 0.98]	0.95 [0.08, 0.95]	0.98 [0.89, 0.99]	0.95 [0.82, 1.00]
NPV	0.98 [0.98, 0.98]	0.97 [0.96, 0.97]	0 [0, 0.98]	0 [0, 0.97]	0.98 [0, 0.99]	0.97 [0, 0.97]	0.99 [0.98, 0.99]	0.96 [0.96, 0.97]
Accuracy	0.98 [0.98, 0.98]	0.96 [0.96, 0.97]	0.08 [0.08, 0.98]	0.08 [0.08, 0.97]	0.98 [0.08, 0.99]	0.97 [0.08, 0.97]	0.99 [0.98, 0.99]	0.96 [0.95, 0.97]
F-1 score	0.86 [0.84, 0.89]	0.74 [0.71, 0.75]	0.15 [0.15, 0.85]	0.15 [0.15, 0.75]	0.89 [0.15, 0.91]	0.74 [0.15, 0.77]	0.92 [0.87, 0.91]	0.72 [0.68, 0.75]
AUPRC	0.94 [0.90, 0.95]	0.80 [0.77, 0.81]	0.88 [0.86, 0.89]	0.81 [0.78, 0.81]	0.91 [0.87, 0.92]	0.79 [0.74, 0.82]	0.92 [0.88, 0.92]	0.78 [0.74, 0.81]

Notes: This table represents the performance of the elastic net model, logistic regression model, random forests, and support vector machines (SVM), which were trained on 80% of the 1,874 reviewed sentences and tested on the remaining 20%. The numbers in brackets present the estimated 95% confidence intervals.

Table 3 represents the characteristics of patients. Table 3 also includes number of patients and the number of clinical notes, which were captured during those visits. Negative descriptors were more frequently used for Black patients (70.5%) compared with White (10.1%) and Other racial groups (19.4%) ( *p* < 0.001). They were also more common among younger patients (18–29 years: 49.3%) and those with Medicare/Medicaid insurance (65.3%). Although negative descriptors were more commonly observed among younger patients in raw percentages, adjusted analysis showed that younger age was associated with lower odds of having a negative descriptor in clinical notes. Similarly, case patients had slightly fewer negative descriptors overall but had higher adjusted odds of having them. Black patients were associated with higher, and commercial insurance with lower, adjusted odds of negative descriptors.

**Table 3:** Patients' characteristics

			Negative patient descriptor in clinical notes				
	All patients with negative keywords ( *n* = 851)		None ( *N* = 407)		One or more ( *N* = 444)		*p*
	%	Number	%	Number	%	Number	
Number of patients	100	851	47.8	407	52.2	444	
Number of clinical notes	100	1384	48.1	665	51.9	719	
Age (y)							
18–29	42.9	365	35.9	146	49.32	219	<0.001
30–44	54.3	462	61. 7	251	47.5	211	
45+	0.71	6	0.98	4	0.45	2	
Race							
Black	60.3	513	49.1	200	70.5	313	<0.001
White	20.2	172	31.2	127	10.1	45	
Other	19.5	166	19.7	80	19.4	86	
Insurance type							
Medicare/Medicaid	53.0	451	39.6	161	65.3	290	<0.001
Commercial	43.9	374	56.8	231	32.2	143	
Self-pay	3.06	26	3.69	15	2.48	11	
Pregnancy type							
Case group	7.52	64	5.9	24	9.01	40	0.11
Control group	92.5	787	94.1	383	90.9	404	

Notes: This table outlines the characteristics of patients who had at least one negative keyword recorded in their clinical notes. The cohort is then divided based on the decision of the elastic net model, indicating whether the negative keyword was classified as a negative patient descriptor or not. The final column on the right presents the p-values comparing the characteristics of patients from the two subgroups.

#### Feature Analysis

To better understand model limitations, we manually examined misclassified sentences (false positives and false negatives) from the held-out test set (20% of annotated sentences). Overall, false negatives occurred more frequently than false positives, indicating that the classifier was conservative in labeling sentences as containing negative descriptors.

False positives most often occurred when negative keywords were used in a nonstigmatizing or negated context. For example, the sentence “no anxiety, depression, or agitation” was incorrectly flagged as a negative descriptor, despite the presence of explicit negation. On the other hand, false negatives often involved more subtle negative phrasing without explicit keywords, or sentences with multiple clauses where the descriptor was de-emphasized. The following negative keywords were involved in false negative cases: “complian,” “agitated,” “aggress,” “cooperat,” “defensive,” “exaggerate,” “noncompliant,” and “refuse.” The most frequent source of false negatives was “complian.” In these cases, clinicians documented that the patient reported compliance with a medication regimen, while simultaneously noting doubt or a history of noncompliance (“patient reports compliance with medications but unclear if this is accurate given her past”). Such nuanced contexts proved difficult for the model to capture. Other false negatives included sentences where patients were described as refusing care, being uncooperative, or exhibiting agitation or defensiveness.

These findings suggest that explicit negation detection and richer contextual modeling (e.g., sentence embeddings or transformer-based approaches) could reduce errors. Importantly, the model's conservative tendency means it likely underestimates, rather than overestimates, the prevalence of negative descriptors in clinical notes.

### Odds Ratio

Table 4 presents the adjusted and unadjusted odds ratio of having a negative descriptor in a patient's clinical notes given specific demographic values and pregnancy risk type. Adjusted and unadjusted odds ratio achieved similar results. Younger patients had lower odds of having a negative descriptor in their clinical notes. Black patients and the patients with Medicare/Medicaid or self-pay insurance type had higher odds of having a negative descriptor in their clinical notes. Being part of the case group increased the odds of having a negative descriptor recorded in a patient's clinical notes. Supplementary Appendix E (available in the online version only) includes the estimated coefficients for the adjusted and unadjusted logistic regression models.

**Table 4:** Odds ratio of having at least one negative descriptor in a patient's clinical notes

Characteristics	Unadjusted odds ratio	Adjusted odds ratio
Age (y; reference: 45+)		
18–29	0.69	0.66 a
30–44	0.52	0.68
Race (reference: White)		
Black	2.66	2.05 d
Other	1.23	0.97
Insurance type (reference: Commercial)		
Medicare/Medicaid	1.88	1.59 d
Self-pay	1.39	1.42
Pregnancy type (reference: control group)		
Case group	1.52	1.40 b

Notes: In calculating the odds ratios, the demographic features and pregnancy risk type were the independent features and having a recorded negative descriptor, which was also detected by the elastic net model, was the dependent binary outcome. Reference value is 1.

a *p* < 0.1.

b *p* < 0.05.

c *p* < 0.01 (none of these *p* -values can be assigned to reference c while reference c needs to stay in this description.).

d *p* < 0.001.

## Discussion

### Racial Bias in Maternal Documentation

Our analysis suggests that negative descriptors are disproportionately used in describing Black and White patients. Similar to the results of the study conducted by Sun et al., Black patients had higher odds of being described with negative descriptors in five types of clinical notes. 27 According to Goddu et al., recorded negative descriptors in patients' clinical notes may negatively affect provider's decision in patient care and result in less aggressive pain management plans. 32 Future studies are needed to identify the effects of recorded negative descriptors in clinical notes on the quality of care that the patient received.

The analysis suggested that maternal patients in the case group and the patients who had Medicare/Medicaid or self-pay insurance were more likely to have a negative descriptor in their EHR clinical notes. On the other hand, the odds of having a negative descriptor were lower for younger patients.

### Importance of Initial Patient Encounter Documentation

Keywords such as “challenging” and “resist” are commonly used by health care providers to describe a patient. 33 34 Although such negative keywords may not be the explicit stigmatizing terms, but they may potentially describe a patient in a negative way to the readers. 33 34 Notably, only 18% of inpatient notes are manually authored; the majority are autopopulated or copied from previously recorded notes, particularly from history and physical documentation. 35 As a result, negative descriptors recorded early in the hospitalization—especially in history and physical notes—may be repeatedly reproduced in subsequent clinical notes, potentially amplifying negative portrayals of the patient throughout the care continuum.

### Sense of Awareness

A provider's implicit biases in documenting negative descriptors in a patient's clinical notes, may affect other health care providers' perception of the patient and their care providing approach. A study indicated the odds of recording negative descriptors are higher in inpatient compared with outpatient settings. 27 This finding highlights the stressful inpatient clinical environments, increased cognitive burden, and time pressure, which puts inpatient health providers at higher risk of using negative descriptors. 36

### Positive Language Recorded in Electronic Health Record

Initially, the study was solely focused on identifying and analyzing negative descriptors recorded in clinical notes recorded at delivery encounters. In the process of reviewing and validating the identified sentences with negative keywords, we encountered multiple uses of the word *pleasant* in providers clinical notes describing patients in a positive way. For instance, “a pleasant 26-year-old female patient.” Future studies are needed to expand the positive keywords and analyze the clinical notes from another angle.

### Limitations and Future Work

Our study has limitations. First, the data came from two birthing hospitals in the same geographic location, limiting generalizability. The developed machine learning classifier should be validated on clinical notes from multiple birthing hospitals across varying geographical locations. Second, this study was conducted on data spanning January 2016 through March 2020, with only a small portion of the data (March 2020) overlapping with the initial period of the coronavirus disease 2019 pandemic. Because this early pandemic period comprised less than 5% of our cohort, we did not include a variable to adjust for the pandemic in our statistical models. Nonetheless, we acknowledge that pandemic-related disruptions in health care delivery may have influenced provider documentation practices. 27 Future studies using data from later phases of the pandemic could more directly examine the relationship between pandemic-related stressors and changes in the use of negative descriptors. Third, this study requires reviewers with expertise in social determinants of health, health disparities, and clinical workflows and operations reviews of the negative keywords identified in the sentences. The required review may introduce reviewer biased to the analysis. Additionally, we did not apply an automated negation detection tool during initial preprocessing. Although our manual review process filtered out negated keyword usage, integrating automated negation detection could enhance scalability and consistency in future implementations. Fourth, our analysis was limited to patients of Black, White, and Other race only. The use of the “Other” category includes any other recorded race in the EHR other than Black or White. We chose this method to ensure there was sufficient power to show significant differences between White patients and those of Other races besides Black race. However, the heterogeneity within the “Other” category may mask differences within the category. In addition, our study did not analyze other demographic factors such as ethnicity, marital status, and primary language of the patient. Additionally, although Medicare and Medicaid were grouped together in our insurance analysis, these populations may differ clinically and socially. Medicare recipients in this cohort were likely younger patients with disabilities, not older adults beyond reproductive age, but further disaggregation was not feasible due to sample size limitations. Fifth, while the machine learning classifier performed well, there is a chance of mislabeling a small proportion of the sentences from the clinical notes. This is in part due to the relatively small, annotated sample used to train the classifier. While our stepwise sampling aimed to ensure sentence diversity and our model achieved strong performance, a larger annotated dataset could further reduce the risk of misclassification bias. Sixth, psychiatric patients were not excluded from this analysis. We focused on maternal patients and psychiatric patients can also be mothers. Finally, our analysis was conducted based on a limited list of negative keywords. While we employed word embeddings to expand the list of negative keywords, a natural extension of this study can be focused on the application of advanced NLP techniques and large language models to identify negative tone in the clinical notes for maternal patients.

Future studies are needed to investigate the use of negative descriptors within subjects (i.e., physicians, nurses, and other health care staff), and how it influences EHR documentation and health care outcome. As patients become more aware and access their recorded EHR data, analyses are needed to assess the effect of open note access on the negative descriptors used by health care providers.

While this study focused on identifying patterns in the use of language that may reflect bias, we recognize that the presence of a “negative descriptor” does not inherently indicate inappropriate or biased documentation. Many of these terms are clinically relevant and may be necessary for effective communication and patient safety. Future work should focus on the contextual appropriateness of language, ideally incorporating temporality, clinical status, and interclinician variation to better distinguish when documentation reflects bias versus necessary clinical observation.

## Conclusion

Utilizing a list of negative keywords and NLP, we analyzed the potentially negative descriptors in clinical notes recorded during delivery encounters. The findings of this study can be incorporated in health care providers' training to minimize the use of negative descriptors in recording EHR data, which may contribute to subsequent adverse maternal outcomes.

## Clinical Relevance Statement

Our study indicates that negative descriptors were disproportionately found in Black patients' clinical notes compared with their White counterparts. This difference may indicate existing implicit racial bias not only among health care providers but also among the broader attitude maintained by the health care team. Moreover, racial implicit biases raise concern about the care provided to historically minoritized groups such as Black patients.

## Multiple-Choice Questions

1. What was the primary objective of the study?
  - To develop a new EHR system for maternal care
  - To identify factors contributing to maternal mortality through laboratory results
  - To examine the association between negative descriptors in EHR notes and adverse maternal outcomes
  - To predict postpartum depression using machine learning
  **Correct Answer** : The correct answer is option c. The study aimed to use natural language processing (NLP) to identify negative patient descriptors in maternal clinical notes and determine whether their presence was linked to adverse outcomes and varied by patient demographics, such as race and insurance type.
2. Which demographic was most frequently associated with negative patient descriptors in the clinical notes?
  - White patients with private insurance
  - Black patients with public insurance
  - Patients over 45 years of age
  - Hispanic patients with commercial insurance
  **Correct Answer** : The correct answer is option b. The study found that negative descriptors appeared disproportionately in the clinical notes of Black patients, particularly those with Medicare or Medicaid coverage. This pattern suggests that implicit bias and social inequities may influence how healthcare providers document patient interactions.
3. What machine learning method was used to classify sentences containing negative descriptors?
  - Support Vector Machine
  - Random Forest
  - Logistic regression with elastic net regularization
  - Naive Bayes
  **Correct Answer** : The correct answer is option c. This method combines LASSO and Ridge regularization, helping handle high-dimensional text data while reducing overfitting and improving model generalization.
4. What was one of the main conclusions of the study?
  - There is no evidence of bias in EHR documentation.
  - The use of negative descriptors was equally distributed across all racial groups.
  - Negative descriptors may reflect implicit bias and disproportionately affect Black patients.
  - Natural language processing cannot reliably identify biased language in EHRs.
  **Correct Answer** : The correct answer is option c.
