# Supplementary material for: A Natural Language Processing Approach to Identify Negative Patient Descriptors in Electronic Health Records for Maternal Care
Source: Appl Clin Inform. 2025 Oct 28;16(5):1475–85. doi: 10.1055/a-2703-7227 (PMC12566921; doi:10.1055/a-2703-7227)
Supplement: Supplementary file 1 — Supplementary Material [file 10-1055-a-2703-7227_27330159.pdf]

Supplementary Materials

Appendix A

Table SA-1. List of 15 initial keywords and their roots which were utilized to expand the keywords list.

Keyword	Root
Non-adherent	Adher
Aggressive	Aggress
Agitated	Agitat
Angry	Angr
Challenging	challeng
Combative	combat
Non-compliant	Complian
Confront	Confront
Non-cooperative/ uncooperative	Cooperat
Defensive	Defens
Exaggerate	Exaggerate
Hysterical	Hysteric
Unpleasant	Pleasant
Refuse	Refus
Resist	Resist

© Thieme. All rights reserved.

Appendix B

Table SB-1. Frequency of the keywords and the similar words identified by Word2Vec model.

Keyword	Frequency count of keyword	Type of word2vec model	Similar words identified by W2V model	Frequency count of similar words	score
adhere	813	CBOW	reiterated	64	0.503312767
adhere	813	CBOW	recipes	4	0.484682679
adhere	813	CBOW	advised	3,708	0.482761353
adhere	813	CBOW	reevaluation	113	0.473314285
adhere	813	CBOW	comply	131	0.472381294
adhere	813	CBOW	counselled	148	0.471404493
adhere	813	CBOW	brat	19	0.470893502
adhere	813	CBOW	apixaban	99	0.468562424
adhere	813	CBOW	strongly	278	0.452957869
adhere	813	SG	reemphasize	8	0.786991298
adhere	813	SG	maximized	7	0.768399537
adhere	813	SG	neruology	3	0.73920691
adhere	813	SG	reiterated	64	0.735331655
adhere	813	SG	inportance	3	0.732097864
adhere	813	SG	humolog	26	0.730142951
adhere	813	SG	antiepileptic	15	0.725264966
adhere	813	SG	complied	4	0.724098861
aggressive	338	CBOW	weaned	185	0.631027341
aggressive	338	CBOW	diuretics	109	0.592749
aggressive	338	CBOW	hfnc	81	0.590752304
aggressive	338	CBOW	remains	1,641	0.590225041
aggressive	338	CBOW	diuresis	396	0.589936137
aggressive	338	CBOW	pressor	65	0.58407867
aggressive	338	CBOW	despite	1,175	0.580281556
aggressive	338	CBOW	decompensated	227	0.575151324
aggressive	338	CBOW	challenging	100	0.570676029
aggressive	338	CBOW	improving	1,485	0.560994625
aggressive	338	SG	remains	1,641	0.729159772
aggressive	338	SG	redirect	25	0.713293433
aggressive	338	SG	escalation	28	0.71069634
aggressive	338	SG	ceeg	5	0.708794296
aggressive	338	SG	flolan	6	0.705578506
aggressive	338	SG	nephrotoxicity	19	0.705429554
aggressive	338	SG	bivad	16	0.702041328
aggressive	338	SG	reeval	17	0.69535327
aggressive	338	SG	inappropriate	66	0.691014171
aggressive	338	SG	hypervolemia	33	0.690297246
agitation	517	CBOW	impulsivity	192	0.728681326
agitation	517	CBOW	aggression	196	0.710090995
agitation	517	CBOW	hopelessness	223	0.696563482
agitation	517	CBOW	anxiety	9,472	0.691328406
agitation	517	CBOW	mania	225	0.687310398
agitation	517	CBOW	psychotic	340	0.634087205
agitation	517	CBOW	ptsd	305	0.620260835
agitation	517	CBOW	inventory	9	0.590317547
agitation	517	CBOW	lability	14	0.571775854
agitation	517	CBOW	insomnia	1,305	0.563346922
agitation	517	SG	propanolol	13	0.735197186
agitation	517	SG	anxiety	9,472	0.734258711
agitation	517	SG	impulsivity	192	0.68848598
agitation	517	SG	ambivalent	22	0.66605109
agitation	517	SG	aggression	196	0.653654337
agitation	517	SG	hopeful	88	0.65327245
agitation	517	SG	fogginess	6	0.647066057
agitation	517	SG	zydis	15	0.644477844
agitation	517	SG	psychosis	384	0.643618345
agitation	517	SG	doxazosin	288	0.641516447

anger	956	CBOW	guilt	63	0.746496916
anger	956	CBOW	frustration	45	0.71363467
anger	956	CBOW	moods	94	0.702495337
anger	956	CBOW	worry	288	0.696990192
anger	956	CBOW	conflicts	55	0.678032398
anger	956	CBOW	criticism	11	0.673403502
anger	956	CBOW	emotional	761	0.672233582
anger	956	CBOW	tearfulness	45	0.662324429
anger	956	CBOW	feelings	696	0.662140429
anger	956	CBOW	cope	67	0.657128513
anger	956	SG	irrational	5	0.819844186
anger	956	SG	suicidality	24	0.79769516
anger	956	SG	frightening	38	0.792833209
anger	956	SG	assertiveness	7	0.791740537
anger	956	SG	criticism	11	0.790568054
anger	956	SG	anhedonia	20	0.784584045
anger	956	SG	resentment	3	0.780231655
anger	956	SG	guilt	63	0.7741974
anger	956	SG	frustration	45	0.770004451
anger	956	SG	alienated	3	0.767386615
challenge	343	CBOW	antiemetics	48	0.662216306
challenge	343	CBOW	restart	504	0.60849905
challenge	343	CBOW	reevaluate	177	0.57971704
challenge	343	CBOW	tonight	440	0.579022527
challenge	343	CBOW	observe	174	0.578619838
challenge	343	CBOW	reglan	754	0.570588291
challenge	343	CBOW	transition	939	0.559885144
challenge	343	CBOW	transitioned	363	0.55827862
challenge	343	CBOW	gcsf	7	0.556519568
challenge	343	CBOW	rocephin	293	0.548475564
challenge	343	SG	tolerate	769	0.719937503
challenge	343	SG	tonight	440	0.719144106
challenge	343	SG	hydrate	211	0.707150102
challenge	343	SG	wsas	3	0.70563525
challenge	343	SG	perocet	7	0.705163538
challenge	343	SG	stalled	8	0.705092847
challenge	343	SG	premedicate	7	0.702284753
challenge	343	SG	ivfs	25	0.702102423
challenge	343	SG	kayexelate	7	0.697708786
challenge	343	SG	bmz	414	0.69749409
combat	51	CBOW	monastat	4	0.565375865
combat	51	CBOW	doxium	3	0.544718146
combat	51	CBOW	hyperdefecation	14	0.539550185
combat	51	SG	minded	3	0.824509919
combat	51	SG	hyperglcyemia	6	0.816760123
combat	51	SG	informs	9	0.812826574
combat	51	SG	rechallenge	7	0.80268693
combat	51	SG	realize	23	0.800851285
compliance	1648	CBOW	compliant	687	0.61426121
compliance	1648	CBOW	arv	162	0.606473386
compliance	1648	CBOW	immunosuppression	604	0.563368499
compliance	1648	CBOW	adherence	440	0.545280635
compliance	1648	CBOW	lifelong	113	0.530567586
compliance	1648	CBOW	noncompliance	163	0.516793787
compliance	1648	CBOW	regimen	2,555	0.493936598
compliance	1648	CBOW	antihypertensive	256	0.485256493
compliance	1648	CBOW	antipsychotic	10	0.479475707
compliance	1648	CBOW	ppi	144	0.476512074
compliance	1648	SG	implementation	5	0.697200656
compliance	1648	SG	poor	2,154	0.692761838
compliance	1648	SG	regimen	2,555	0.687959075
compliance	1648	SG	prognosis	76	0.678895772
compliance	1648	SG	reemphasize	8	0.670022428
compliance	1648	SG	endari	17	0.666971862
compliance	1648	SG	willl	8	0.666873693
compliance	1648	SG	compliant	687	0.664361238

cooperative	5839	CBOW	aaox	1,021	0.695072234
cooperative	5839	CBOW	affect	9,229	0.667125225
cooperative	5839	CBOW	oriented	19,899	0.634528399
cooperative	5839	CBOW	calm	621	0.629436255
cooperative	5839	CBOW	psych	7,792	0.618732512
cooperative	5839	CBOW	normocephalic	5,719	0.590999186
cooperative	5839	CBOW	orientedx	31	0.578015327
cooperative	5839	CBOW	smiling	37	0.565413475
cooperative	5839	CBOW	attentive	137	0.55953449
cooperative	5839	CBOW	conversational	10	0.553472579
cooperative	5839	SG	cheerful	12	0.772691905
cooperative	5839	SG	affect	9,229	0.769461989
cooperative	5839	SG	blunted	11	0.763661563
cooperative	5839	SG	karnofsky	5	0.759842694
cooperative	5839	SG	behaviour	3	0.757000208
cooperative	5839	SG	conversive	4	0.750609338
confront	42	CBOW	magnitude	8	0.536139846
confront	42	CBOW	regret	20	0.532719254
defense	104	CBOW	mistakenly	16	0.817211211
defense	104	CBOW	produces	34	0.744777501
defense	104	CBOW	sid	31	0.729338467
defense	104	CBOW	enters	74	0.722278416
defense	104	CBOW	dermatophytosis	36	0.721312106
defense	104	CBOW	absorbs	5	0.710776746
defense	104	CBOW	especiall	28	0.701643527
defense	104	CBOW	sensitize	3	0.692959547
defense	104	CBOW	fight	117	0.690311134
defense	104	SG	weakens	5	0.800207496
defense	104	SG	overactive	16	0.766280413
defense	104	SG	fight	117	0.75023365
defense	104	SG	adenoviruses	6	0.736219406
defense	104	SG	overreact	6	0.734675288
defense	104	SG	weakened	54	0.732729554
defense	104	SG	mistakenly	16	0.725964189
defense	104	SG	alters	4	0.722094178
defense	104	SG	reacts	11	0.714493155
defense	104	SG	weaken	31	0.714420557
exaggerate	14	CBOW	sideswiped	6	0.455234796
exaggerate	14	CBOW	translating	4	0.445488602
exaggerate	14	CBOW	noncardiac	4	0.440858364
exaggerate	14	CBOW	reach	9,770	0.426804304
exaggerate	14	CBOW	haemolysis	16	0.421945035
exaggerate	14	CBOW	anginal	131	0.415411443
exaggerate	14	CBOW	preppin	50	0.411320448
exaggerate	14	CBOW	silhouette	370	0.407399267
exaggerate	14	SG	crowding	3	0.871299982
exaggerate	14	SG	pefr	3	0.856892586
hysterical	5	CBOW	proud	4	0.599112332
hysterical	5	SG	burnout	4	0.860323668
pleasant	840	CBOW	nad	3,266	0.759144187
pleasant	840	CBOW	conversant	104	0.718060911
pleasant	840	CBOW	smiling	37	0.705505908
pleasant	840	CBOW	nourished	6,732	0.680666327
pleasant	840	CBOW	talkative	19	0.655956328
pleasant	840	CBOW	obese	1,771	0.64962858
pleasant	840	CBOW	wdwn	47	0.635644019
pleasant	840	CBOW	aaox	1,021	0.631968737
pleasant	840	CBOW	conversational	10	0.614192724
pleasant	840	CBOW	groomed	75	0.608538806
pleasant	840	SG	vore	9	0.755310476
pleasant	840	SG	conversant	104	0.754234433
pleasant	840	SG	morbidly	163	0.735719919
pleasant	840	SG	conversational	10	0.730676293
pleasant	840	SG	oveerall	4	0.727593601
pleasant	840	SG	obsese	11	0.721567094
pleasant	840	SG	conversive	4	0.716795087

pleasant	840	SG	groomed	75	0.712587476
refused	1730	CBOW	refusing	422	0.713872254
refused	1730	CBOW	dosage	1,180	0.505454659
refused	1730	CBOW	dillon	723	0.473208517
refused	1730	CBOW	medroxyproge	22	0.463348746
refused	1730	CBOW	mason	495	0.462760955
refused	1730	CBOW	lapek	312	0.462552756
refused	1730	CBOW	barry	638	0.459652871
refused	1730	CBOW	balaker	1,309	0.458863974
refused	1730	SG	refusing	422	0.804847538
refused	1730	SG	afternoonb	3	0.692557991
refused	1730	SG	refuses	326	0.678466797
refused	1730	SG	declining	166	0.677189589
refused	1730	SG	claimes	3	0.676993608
refused	1730	SG	declined	2,318	0.676352262
refused	1730	SG	ekema	5	0.65455687
refused	1730	SG	hemmorage	4	0.649799168
refused	1730	SG	requests	1,759	0.647903681
resistant	735	CBOW	susceptible	150	0.774208665
resistant	735	CBOW	aerogenes	263	0.765785575
resistant	735	CBOW	methicillin	28	0.765309691
resistant	735	CBOW	enterobacter	230	0.75535059
resistant	735	CBOW	faecium	180	0.75226599
resistant	735	CBOW	carbapenem	114	0.747135103
resistant	735	CBOW	klebsiella	624	0.738275468
resistant	735	CBOW	kleb	112	0.735532165
resistant	735	CBOW	coli	969	0.729816854
resistant	735	CBOW	citrobacter	85	0.72066468
resistant	735	SG	enterobactor	5	0.788323224
resistant	735	SG	carbapenem	114	0.737603366
resistant	735	SG	multidrug	29	0.732683361
resistant	735	SG	relapsed	8	0.729931891
resistant	735	SG	mics	14	0.726924062
resistant	735	SG	ceftraixone	4	0.72091043
resistant	735	SG	deescalated	8	0.71334219
resistant	735	SG	enterobacteriacae	8	0.710986555

Table SB-2. Frequency of the keywords and the similar words identified by Word2Vec model which were validated by our subject matter experts.

Keyword	Frequency count of keyword among EHR clinical notes	Type of word2vec model	Similar words identified by W2V model	Frequency count of similar words	score
adhere	813	CBOW	reiterated	64	0.503312767
adhere	813	CBOW	reevaluation	113	0.473314285
adhere	813	CBOW	comply	131	0.472381294
adhere	813	CBOW	counselled	148	0.471404493
adhere	813	CBOW	brat	19	0.470893502
adhere	813	CBOW	strongly	278	0.452957869
adhere	813	SG	reemphasize	8	0.786991298
adhere	813	SG	reiterated	64	0.735331655
adhere	813	SG	complied	4	0.724098861
aggressive	338	CBOW	despite	1,175	0.580281556
aggressive	338	CBOW	challenging	100	0.570676029
aggressive	338	CBOW	improving	1,485	0.560994625
aggressive	338	SG	remains	1,641	0.729159772
aggressive	338	SG	redirect	25	0.713293433
aggressive	338	SG	escalation	28	0.71069634
aggressive	338	SG	reeval	17	0.69535327
aggressive	338	SG	inappropriate	66	0.691014171
agitation	517	CBOW	impulsivity	192	0.728681326
agitation	517	CBOW	aggression	196	0.710090995
agitation	517	CBOW	hopelessness	223	0.696563482
agitation	517	CBOW	anxiety	9,472	0.691328406
agitation	517	CBOW	mania	225	0.687310398
agitation	517	CBOW	psychotic	340	0.634087205
agitation	517	CBOW	ptsd	305	0.620260835
agitation	517	CBOW	lability	14	0.571775854
agitation	517	SG	anxiety	9,472	0.734258711
agitation	517	SG	impulsivity	192	0.68848598
agitation	517	SG	aggression	196	0.653654337
agitation	517	SG	fogginess	6	0.647066057
agitation	517	SG	psychosis	384	0.643618345
anger	956	CBOW	guilt	63	0.746496916
anger	956	CBOW	frustration	45	0.71363467
anger	956	CBOW	moods	94	0.702495337
anger	956	CBOW	worry	288	0.696990192
anger	956	CBOW	conflicts	55	0.678032398
anger	956	CBOW	criticism	11	0.673403502
anger	956	CBOW	emotional	761	0.672233582
anger	956	CBOW	tearfulness	45	0.662324429
anger	956	CBOW	feelings	696	0.662140429
anger	956	CBOW	cope	67	0.657128513
anger	956	SG	irrational	5	0.819844186
anger	956	SG	suicidality	24	0.79769516
anger	956	SG	frightening	38	0.792833209
anger	956	SG	assertiveness	7	0.791740537
anger	956	SG	criticism	11	0.790568054
anger	956	SG	resentment	3	0.780231655
anger	956	SG	guilt	63	0.7741974
anger	956	SG	frustration	45	0.770004451
anger	956	SG	alienated	3	0.767386615
challenge	343	CBOW	reevaluate	177	0.57971704
challenge	343	SG	tolerate	769	0.719937503
combat	51	SG	rechallenge	7	0.80268693
combat	51	SG	realize	23	0.800851285
compliance	1648	CBOW	compliant	687	0.61426121
compliance	1648	CBOW	noncompliance	163	0.516793787
compliance	1648	SG	poor	2,154	0.692761838
compliance	1648	SG	reemphasize	8	0.670022428
compliance	1648	SG	compliant	687	0.664361238
cooperative	5839	CBOW	calm	621	0.629436255
cooperative	5839	CBOW	psych	7,792	0.618732512

 © Thieme. All rights reserved.

cooperative	5839	CBOW	smiling	37	0.565413475
cooperative	5839	CBOW	attentive	137	0.55953449
cooperative	5839	CBOW	conversational	10	0.553472579
cooperative	5839	SG	cheerful	12	0.772691905
cooperative	5839	SG	blunted	11	0.763661563
cooperative	5839	SG	behaviour	3	0.757000208
confront	42	CBOW	regret	20	0.532719254
defense	104	CBOW	mistakenly	16	0.817211211
defense	104	CBOW	sensitize	3	0.692959547
defense	104	CBOW	fight	117	0.690311134
defense	104	SG	weakens	5	0.800207496
defense	104	SG	overactive	16	0.766280413
defense	104	SG	fight	117	0.75023365
defense	104	SG	overreact	6	0.734675288
defense	104	SG	weakened	54	0.732729554
defense	104	SG	mistakenly	16	0.725964189
defense	104	SG	reacts	11	0.714493155
defense	104	SG	weaken	31	0.714420557
hysterical	5	CBOW	proud	4	0.599112332
hysterical	5	SG	burnout	4	0.860323668
pleasant	840	CBOW	conversant	104	0.718060911
pleasant	840	CBOW	smiling	37	0.705505908
pleasant	840	CBOW	talkative	19	0.655956328
pleasant	840	CBOW	obese	1,771	0.64962858
pleasant	840	CBOW	conversational	10	0.614192724
pleasant	840	CBOW	groomed	75	0.608538806
pleasant	840	SG	conversant	104	0.754234433
pleasant	840	SG	conversational	10	0.730676293
pleasant	840	SG	obsese	11	0.721567094
pleasant	840	SG	conversive	4	0.716795087
refused	1730	CBOW	refusing	422	0.713872254
refused	1730	SG	refusing	422	0.804847538
refused	1730	SG	refuses	326	0.678466797
refused	1730	SG	declining	166	0.677189589
refused	1730	SG	claimes	3	0.676993608
refused	1730	SG	declined	2,318	0.676352262
resistant	735	CBOW	susceptible	150	0.774208665
resistant	735	SG	deescalated	8	0.71334219

Appendix C

Table SC-1. Examples of Negative Keywords. These examples of keywords were validated by our subject matter experts in conveying a negative patient description.

Keyword	Example of sentence with negative keyword
refuse	given advanced gestational age, recommend iol, patient **refuses**.
aggressive	transferred to inpatient psychiatry after displaying irritable mood and **aggression** towards staff.
noncompliant	patient has been **noncompliant** with nonpharmaceutical therapy - has not been careful with diet.
agitated	upon further interview with [Dr] entering the room, the patient became very angry and **agitated** stating that she always has chest pain, was having it currently, and that no one ever does anything about it and that she was unhappy with her care at hospital center.
challenging	attending physician impressions: **challenging** case
combative	she was brought back in the ED, confused, agitated, **combative**, and yelling. she required chemical and physical restraints again.
nonadherent	patient has also been **nonadherent** to medications (Flonase and Claritin). there is also likely a psychiatric component of symptoms with anxiety and depression.
defensive	when I asked patient about her utox being positive she became **defensive** and upset and said it was because of Tylenol with codeine -this might not explain how baby has utox positive as patient claims she had only one dose.
hysterical	patient gets **hysterical** and worries that worker to hold her down, but I am assuring her that that is not true.
uncooperative	tender to palpation in lower pelvis/suprapubic area, **uncooperative** with exam, pushing

 © Thieme. All rights reserved.

Appendix D

Table SD-1. Selected features from unstructured free-text clinical notes and the associated estimated coefficient from negative keywords classifiers.

Words	Estimated Coeff
'admit',	0.17932089
'affect',	0
'agit',	0.275931856
'alert',	-0.081105979
'appropri',	-0.240924744
'atraumat',	-0.024486577
'calf',	-0.129656616
'challeng',	-0.161123951
'complianc',	-0.249403915
'compliant',	-0.21800988
'cooper',	-0.167631674
'cooperative',	-0.200717365
'deficit',	-0.009237079
'distress',	-0.081740109
'doctor',	0.056659132
'edema',	-0.073272291
'exam',	0.013452311
'extremities',	-0.027825013
'focal',	-0.058292486
'headface',	-0.024831379
'histori',	0.043196781
'intact',	-0.181834607
'lesion',	0
'medic',	0.205876518
'mood',	0.177322457
'noncompli',	0.882793328
'normocephal',	-0.029848998
'pleasant',	-0.221143818
'present',	0.153534133
'psych',	-0.058391699
'psychiatric',	0.086232192
'rash',	0
'refus',	0.847597991
'report',	-0.006260319
'resist',	-0.186751342
'skin',	-0.069250051
'tender',	0
'thought',	-0.239448309
'time',	0.219241457
'uti'	-0.178846277

Appendix E

Table SE-1. Estimated coefficients associated with adjusted and unadjusted logistic regression models to predict the odds ratios of having a negative descriptor in a patient's clinical notes.

Characteristics	Unadjusted estimated Coefficient	Adjusted Estimated Coefficient
Age		
18-29	-0.22	-0.24
30-44	-0.31	-0.25
Race		
Black	0.15	0.07
Other	-0.19	-0.26
Insurance		
Medicare/ Medicaid	0.13	0.13
Self pay	0.11	0.12
Pregnancy Type		
Case group	-0.16	-0.19

Applied Clinical Informatics | DOI 10.1055/a-2703-7227 | © Thieme. All rights reserved.
